# Supplementary material for: What is the evidence for efficacy, effectiveness and safety of surgical interventions for plantar fasciopathy? A systematic review
Source: PLoS One. 2022 May 18;17(5):e0268512. doi: 10.1371/journal.pone.0268512 (PMC9116678; doi:10.1371/journal.pone.0268512)
Supplement: S1 Appendix — (DOCX) [file pone.0268512.s002.docx]

**ONLINE SUPPLEMENTARY FILE**

**Appendix 1: Medline Search Strategy**

(Plantar fasci* OR heel pain OR policeman* heel OR heel spur OR painful heel syndrome OR baxter* neuropath* OR plantar neuropath* OR calcaneal neuropath* OR nerve entrapment OR Calcaneodynia)

AND

(Plantar fascia release OR Endoscopic OR gastrocnemius release OR gastrocnemius recession OR PMGR OR spur removal OR drilling OR release OR microfasciotomy OR Surg* OR Fasciotomy OR fasciectomy OR Operat* OR Minimally Invasive OR MIS OR debridement OR microdebride* OR radiofrequency)

We used the filter for RCTs where available in each database. Where unavailable we used the following search terms to identify RCTs: (randomi*ed controlled trial OR controlled clinical trial OR randomi*ed OR placebo OR randomly OR trial OR groups).
