# Supplementary material for: What is the evidence for efficacy, effectiveness and safety of surgical interventions for plantar fasciopathy? A systematic review
Source: PLoS One. 2022 May 18;17(5):e0268512. doi: 10.1371/journal.pone.0268512 (PMC9116678; doi:10.1371/journal.pone.0268512)
Supplement: S2 Appendix — (DOCX) [file pone.0268512.s003.docx]

**ONLINE SUPPLEMENTARY FILE**

**Appendix 2: Assessment of risk of bias in included studies**

We assessed the following for each study:

*Random sequence generation* (checking for possible selection bias). We assessed the method used to generate the allocation sequence as: low risk of bias (any truly random process, e.g. random number table; computer random number generator); unclear risk of bias (method used to generate sequence not clearly stated); high risk of bias (studies using a non-random process (e.g. odd or even date of birth; hospital or clinic record number).

*Allocation concealment* (checking for possible selection bias). The method used to conceal allocation to group prior to assignment determines whether intervention allocation could have been foreseen in advance of, or during recruitment, or changed after assignment. We assessed the methods as: low risk of bias (e.g. telephone or central randomisation; consecutively numbered sealed opaque envelopes); unclear risk of bias (method not clearly stated); high risk of bias (studies that did not conceal allocation (e.g. open list)).

*Blinding of participants:* low risk of bias (participants blinded to allocated intervention; and unlikely that blinding broken); unclear risk of bias (insufficient information to permit judgement of low/high risk of bias); high risk of bias (patients not blinded to allocated intervention OR patients blinded to allocated intervention but it was likely that blinding may have been broken (and a given outcome was likely to be influenced by lack of blinding).

*Blinding of care providers:* low risk of bias (care provider blinded to allocated intervention; and unlikely that blinding broken OR no/incomplete blinding but judged that a given outcome unlikely to be influenced by lack of blinding); unclear risk of bias (insufficient information to permit judgement of low/high risk of bias); high risk of bias (care provider not blinded to allocated intervention and the two interventions clearly identifiable to the care provider as experimental and control OR care provider blinded to allocated intervention but likely that blinding may have been broken (and a given outcome is likely to be influenced by lack of blinding)).

*Blinding of assessor:* low risk of bias (outcome assessor (including patients with respect to self-report outcomes) blinded to patients allocated intervention; and unlikely that blinding broken, or no/incomplete blinding but judged that a given outcome unlikely to be influenced by lack of blinding); unclear risk of bias (insufficient information to permit judgement of low/high risk of bias); high risk of bias (outcome assessor (including patients with respect to self-report outcomes) un-blinded to patients allocated intervention OR outcome assessor blinded to allocated intervention but likely that blinding may have been broken (and a given outcome is likely to be influenced by lack of blinding).

*Incomplete outcome data (drop outs).* We first checked for possible attrition bias by considering if participant drop-out rate was appropriately described and acceptable. Low: if less than 20% drop out and appears to be missing at random. Numbers given per group and reasons for drop out described. Unclear: if less than 20% but reasons not described and numbers per group not given. Unclear that data is missing at random. High: if over 20% even if imputed appropriately.

*Incomplete outcome data (protocol violations).* We separately considered if participants were analysed in the group to which they were allocated. Low: if analysed data in group to which originally assigned (be that with appropriately imputed data or an available case analysis) Unclear: insufficient information provided to determine if analysis was per protocol or intention to treat. High: if per protocol analysis used. Where available data was not analysed or participant’s data was included in group they were not originally assigned to.

*Selective reporting.* We assessed whether studies were free of the suggestion of selective outcome reporting. Methods were assessed as: low risk of bias (study protocol available and all pre-specified outcomes of interest adequately reported. Study protocol not available but all expected outcomes of interest adequately reported. All primary outcomes numerically reported with point estimates and measures of variance for all time points); high risk of bias (incomplete reporting of pre-specified outcomes. One or more primary outcomes was reported using measurements, analysis methods or subsets of data that were not pre-specified. One or more reported primary outcomes were not pre-specified. One or more outcomes of interest reported incompletely and could not be entered into a meta-analysis. Results for a key outcome expected to have been reported excluded).

*Other sources of bias.* We considered other risk factors such as whether trials were stopped early, differences between groups at baseline, timing of outcome assessment, control of co-interventions and author source of funding declarations.
