# Supplementary material for: What is the evidence for efficacy, effectiveness and safety of surgical interventions for plantar fasciopathy? A systematic review
Source: PLoS One. 2022 May 18;17(5):e0268512. doi: 10.1371/journal.pone.0268512 (PMC9116678; doi:10.1371/journal.pone.0268512)
Supplement: S3 Appendix — (DOCX) [file pone.0268512.s004.docx]

**ONLINE SUPPLEMENTARY FILE**

**Appendix 3: Certainty of the evidence**

Factors that may decrease the certainty of the evidence are:

- study design and risk of bias (downgraded if more than 25% of the participants are from studies with a high risk of bias);
- inconsistency of results (downgraded if significant heterogeneity is present by visual inspection or if the I^2^ value was greater than 50%);
- indirectness (generalisability of the findings; downgraded if more than 50% of the participants are outside the target group);
- imprecision (downgraded if fewer than 400 participants are included in the comparison for continuous data or there are fewer than 300 events for dichotomous data [1] and other factors (e.g. reporting bias, publication bias)).

The GRADE system uses the following criteria for assigning grade of evidence:

- High-certainty: we are very confident that the true effect lies close to that of the estimate of the effect;
- Moderate-certainty: we are moderately confident in the effect estimate; the true effect is likely to be close to the estimate of effect, but there is a possibility that it is substantially different;
- Low-certainty: our confidence in the effect estimate is limited; the true effect may be substantially different from the estimate of the effect;
- Very low certainty: we have very little confidence in the effect estimate; the true effect is likely to be substantially different from the estimate of effect.

**Reference**

1. Mueller PS, Montori VM, Bassler D, et al. Ethical issues in stopping randomized trials early because of apparent benefit. *Ann Intern Med* 2007;146:878–881.
