# Supplementary material for: What is the evidence for efficacy, effectiveness and safety of surgical interventions for plantar fasciopathy? A systematic review
Source: PLoS One. 2022 May 18;17(5):e0268512. doi: 10.1371/journal.pone.0268512 (PMC9116678; doi:10.1371/journal.pone.0268512)
Supplement: S4 Appendix — (DOCX) [file pone.0268512.s005.docx]

**ONLINE SUPPLEMENTARY FILE**

**Appendix 4: Characteristics of excluded trials and trials awaiting classification.**

|  | **Excluded trials:** | **Reason for exclusion** |
| --- | --- | --- |
| 1 | Al-Ashhab et al. (2018) | Not RCT |
| 2 | Cottom et al. (2016) | Not RCT |
| 3 | Duan, Chen (2016) | No full text available |
| 4 | Landsman et al. (2013) | Not surgery |
| 5 | Li et al. (2014) | Not surgery |
| 6 | Othman, Ragab. (2010) | Not RCT |
| 7 | Ozan et al. (2017) | Not RCT |
| 8 | Saxena et al. (2013) | Not RCT |
| 9 | Tang et al. (2020) | Not RCT |
| 10 | Wu et al.(2017) | Not surgical |
| 11 | Ye et al.(2015) | Not surgical |
| 12 | National Clinical Trial number: 02287714 | Trial withdrawn due to ‘low(no) recruitment’ |

|  | **Trials awaiting classification** | **Reason** |
| --- | --- | --- |
| 1 | Plantar fasciosis treatment using coblation  *National Clinical Trial number: NCT00189592* | Study registered as ‘completed’, unable to locate published trial. No response to email contact. |
| 2 | Post-operative Strength of Plantar flexion after Endoscopic Isolated Gastrocnemius versus Combined Gastro-soleus Recession in Patients with Isolated Gastrocnemius Contracture: a Randomized Prospective Comparative Study *Thai Clinical Trials Registry Number: 20180212001* | Study registered as ‘ongoing’, unable to locate published trial. No response to email contact. |
| 3 | Operative versus non-operative treatment of recalcitrant Plantar Fasciitis – a randomised study. *National Clinical Trial number: NCT05066919* | Study registered as ‘not yet recruiting’ |

**References of excluded studies:**

Al-Ashhab M, Elbegawy H, Hasan H. Endoscopic Plantar Fasciotomy Through Two Medial Portals for the Treatment of Recalcitrant Plantar Fasciopathy. *J Foot Ankle Surg* 2018;57:264-268.

Cottom JM, Maker JM, Richardson P, et al. Endoscopic Debridement for Treatment of Chronic Plantar Fasciitis: An Innovative Technique and Prospective Study of 46 Consecutive Patients. *The Journal of Foot and Ankle Surgery.* 2016;55:748-52.

Duan H, Chen SY. Observations on efficacy of small needle scalpel under ultrasonography guidance in plantar fasciitis. *Zhongguo Gu Shang* 2016;29:1092-1096.

Landsman AS, Catanese DJ, Wiener SN,et al. A prospective, randomized, double-blinded study with crossover to determine the efficacy of radio-frequency nerve ablation for the treatment of heel pain. *J Am Podiatr Med Assoc.* 2013;103:8-15

Li S, Shen T, Liang Y, et al. Miniscalpel-Needle versus Steroid Injection for Plantar Fasciitis: A Randomized Controlled Trial with a 12-Month Follow-Up. *Evidence-Based Complementary and Alternative Medicine*. 2014;Article ID 164714.

Othman AM, Ragab EM. Endoscopic plantar fasciotomy versus extracorporeal shock wave therapy for treatment of chronic plantar fasciitis. *Arch Orthop Trauma Surg.* 2010;130:1343-7.

Ozan F, Koyuncu Ş, Gürbüz K, et al. Radiofrequency Thermal Lesioning and Extracorporeal Shockwave Therapy: A Comparison of Two Methods in the Treatment of Plantar Fasciitis. *Foot Ankle Spec*. 2017;10:204-209.

Saxena A, Fournier M, Gerdesmeyer L, et al. Comparison between extracorporeal shockwave therapy, placebo ESWT and endoscopic plantar fasciotomy for the treatment of chronic plantar heel pain in the athlete. *Muscles Ligaments Tendons J*. 2013;2:312-6.

Tang Y, Deng P, Wang G, et al. The Clinical Efficacy of Two Endoscopic Surgical Approaches for Intractable Plantar Fasciitis. *The Journal of Foot and Ankle Surgery* 2020;59:280-285.

Wu YT, Chang CY, Chou YC, et al. Ultrasound-Guided Pulsed Radiofrequency Stimulation of Posterior Tibial Nerve: A Potential Novel Intervention for Recalcitrant Plantar Fasciitis. *Arch Phys Med Rehabil*. 2017;98:964-970.

Ye L, Mei Q, Li M, et al. A comparative efficacy evaluation of ultrasound-guided pulsed radiofrequency treatment in the gastrocnemius in managing plantar heel pain: a randomized and controlled trial. *Pain Med*. 2015;16:782-90.

**References of withdrawn trials:**

NCT02287714. Instep Plantar Fasciotomy With and Without Gastrocnemius Recession. <https://clinicaltrials.gov/ct2/show/NCT02287714>

**References of potentially eligible trials from trial registers:**

NCT00189592. Plantar fasciosis treatment using coblation. https://clinicaltrials.gov/ct2/show/NCT00189592

TCTRN 20180212001. Post-operative Strength of Plantar flexion after Endoscopic Isolated Gastrocnemius versus Combined Gastro-soleus Recession in Patients with Isolated Gastrocnemius Contracture: a Randomized Prospective Comparative Study <http://www.thaiclinicaltrials.org/>

NCT05066919 Operative versus non-operative treatment of recalcitrant Plantar Fasciitis – a randomised study.

https://clinicaltrials.gov/ct2/show/record/NCT05066919
