# Supplementary material for: What is the evidence for efficacy, effectiveness and safety of surgical interventions for plantar fasciopathy? A systematic review
Source: PLoS One. 2022 May 18;17(5):e0268512. doi: 10.1371/journal.pone.0268512 (PMC9116678; doi:10.1371/journal.pone.0268512)
Supplement: S5 Appendix — (DOCX) [file pone.0268512.s006.docx]

**ONLINE SUPPLEMENTARY FILE**

**Appendix 5: Characteristics of the included studies**

Setting

The included trials were conducted in a variety of locations including: Turkey;[24,32] Spain;[29,30] Denmark;[31] Norway;[25] and Egypt.[26-28] For the majority of studies, the setting was unclear, although were likely performed in a secondary care orthopaedic hospital setting.[24,26-30, 32] One study was conducted in a private rheumatology clinic and a University clinic;[31] and one study at a single university hospital.[25]

Participants

The number of participants included per trial in the eight studies ranged from 30-65, with a total of 345 participants with PF randomised. Diagnostic criteria for PF varied between studies and included: clinical diagnosis alone [24,25,27,29,30] or clinical and radiological diagnosis.[28,31,32] The diagnostic criteria in one study was not described.[26]

The duration of symptoms required for inclusion ranged from at least three months to at least 12 months. Seven studies reported baseline VAS for pain.[24,26,27,29-31] First step pain levels ranged from means of 6.1- 7.1 [31] and medians of 6.8-7.1 [27] (scale: 0-10, 0 = no pain). Levels of pain for other reported measures ranged from means of 5.0-8.28 (scale: 0-10, 0 = no pain) [24,26,29-31] and medians of 6.8- 7.1.[25] Two studies did not report baseline pain levels.[28,32]

Two studies included participants with unilateral symptoms.[26,27] One study included participants with unilateral and bilateral symptoms.[31] In five studies it was unclear whether participants had uni-or bilateral symptoms.[24,25, 28-30,32] Seven studies specified that participants had not responded to conservative treatment prior to entering the trial.[24-30,32] It was unclear in one study whether participants had previously trialled conservative management.[31]

Follow-up period

The follow-up period for five of the studies was 12 months;[24-26,29,30,32], one study had a follow-up period of 24 months.[31] For two of the studies,[27,28] the exact follow-up period was unclear and appeared to vary between participants.
