# Supplementary material for: What is the evidence for efficacy, effectiveness and safety of surgical interventions for plantar fasciopathy? A systematic review
Source: PLoS One. 2022 May 18;17(5):e0268512. doi: 10.1371/journal.pone.0268512 (PMC9116678; doi:10.1371/journal.pone.0268512)
Supplement: S6 Appendix — (DOCX) [file pone.0268512.s007.docx]

**ONLINE SUPPLEMENTARY FILE**

**Appendix 6: Risk of bias in the included studies**

**Allocation**

Only one trial reported using, or were judged to have used both adequate sequence generation and allocation concealment [27] and hence was judged as being at low risk of selection bias. For seven trials the risk of selection bias was ‘unclear’ [24-26,28-32] where the methods described to generate allocation sequence or allocation concealment were not clearly reported.

**Blinding**

We rated all studies at high risk of bias on this domain. Reasons for high risk of bias in all studies was a lack of blinding of clinicians. In addition, high risk of bias was present for a lack of blinding of participants in six studies,[25-27,29-32] and for inadequate blinding of assessors in three studies.[25,29,30,32] We deemed three studies to have ‘high’ risk of bias due to inadequate blinding of assessors, clinicians and participants.[25,29,30,32] We noted that in all studies it would not have been possible to blind the surgeons conducting the procedures. For most studies, it would not have been possible to blind participants to the intervention. However, participant and assessor blinding was potentially possible in two studies,[24,28] both deemed to be of ‘unclear’ risk of bias, who compared surgical techniques with similar incision sites, and hence similar between-group surgical scars.

**Incomplete outcome data**

We judged four trials as being of ‘low’ risk of bias on this domain.[24,25,27,31] In four trials, we judged the risk of bias as ‘unclear’ due to either a drop-out rate of less than 20% but reasons or numbers per group were not provided, or, that insufficient information was provided to determine if analysis was per protocol or intention to treat.[26,28-30,32] No studies were judged to be at ‘high’ risk of attrition bias.

**Selective reporting**

We judged three trials to be of ‘high’ risk of reporting bias [26,28-30] due to the absence of clearly reported endpoints,[28] and incomplete reporting of primary or secondary outcomes.[26,29,30] We judged one study to have an ‘unclear’ risk of bias due to a study protocol or trial registration not being available.[32] We contacted the authors of one paper [31] due to incomplete reporting of outcome measure - the authors responded providing the additional data requested, hence, we judged the study to have a ‘low risk of bias’. In the remaining three trials we deemed outcome data reporting appropriate and hence we judged these trials to have a ‘low’ risk of reporting bias.[24,25,27]

**Other potential sources of bias**

We judged two trials to be of ‘high’ risk of other potential sources of bias; one trial due to substantial differences in baseline pain levels between groups which were unadjusted in the analysis;[31] and one trial due to the absence of reporting of a post-operative protocol for the surgical group but the inclusion of a post-procedural rehabilitation protocol for the non-surgical group [26] - this between group difference in post-procedure protocols may have had an impact on the results obtained. We judged one trial to have an ‘unclear’ risk of other potential sources of bias due to the absence of reporting of baseline symptom severity, duration and gender per group.[28] We deemed the five remaining trials to be of ‘low’ risk of other potential sources of bias.
