# Supplementary material for: What is the evidence for efficacy, effectiveness and safety of surgical interventions for plantar fasciopathy? A systematic review
Source: PLoS One. 2022 May 18;17(5):e0268512. doi: 10.1371/journal.pone.0268512 (PMC9116678; doi:10.1371/journal.pone.0268512)
Supplement: S7 Appendix — (DOCX) [file pone.0268512.s008.docx]

**ONLINE SUPPLEMENTARY FILE**

**Appendix 7: Effects of interventions: Secondary Outcome measures**

Endoscopic deep fascial approach fasciotomy versus endoscopic superficial fascial approach fasciotomy [23]

*Secondary Outcomes*

*Function*

Post-operative AOFAS-AHS scores were greater than pre-operative scores for both groups at short, mid and long-term follow-up. There were no clear between group differences at short (effect size [95% CI]: -3.94, [-10.34, 2.46]), mid- (effect size [95% CI]: -2.55, [-8.78, 3.68]) and long-term (effect size [95% CI]: -1.99, [-7.75, 3.77]) follow-up.

*Pain*

It was unclear which pain descriptor was assessed in this study, hence we judged the outcome to be a secondary outcome. Pain scores were lower in the SFA group compared to the DFA group at short term follow up (effect size [95% CI]: 0.65 [-0.34, -1.64]), however there was no clear between group differences in pain at mid- (effect size [95% CI]: 0.34, [-0.63, 1.31]) or long-term (effect size [95% CI]: 0.31, [-0.63, 1.06]) follow-up.

PMGR and stretching versus stretching.[24]

*Secondary Outcomes*

*Pain – worst pain in 24 hours*

In the PMGR group at 3 months the median pain score (VAS worst pain in 24 hours) was 3.3 (range 0-8.1) compared to 6.9 (range 2.1-10) in the stretching only group. At 12 months the median pain score (VAS worst pain in 24 hours) in the PMGR group was 2.8 (range 0-8.1) compared to 7.4 (0.2-9.3) in the stretching only group.

*Function*

In the PMGR group at 3 months the median function score (AOFAS-AHS) was 85.5 (range 64-100) compared to 66.5 (range 36-85) in the stretching only group. At 12 months, the median function score (AOFAS-AHS) in the PMGR group was 88.0 (range 50-100) compared to 65.5 (31-88) in the stretching only group.

*Quality of life*

We were unable to calculate effect sizes as authors reported medians and range only for SF36 subscales. Between group p-values were reported at baseline and 12 months. At twelve months, the authors reported statistical significance for the operative group for all domains of SF36. We noted, however, that the reported data for the ‘role emotion’ domain, suggesting a between group difference, appears to be an error of reporting as the median and range data for this outcome are identical in both groups.

Endoscopic plantar fasciotomy versus platelet rich plasma injection.[25]

*Secondary Outcomes*

*Function*

Both groups demonstrated improvements in function over time; there were no between-group differences ‘over-time’ for the AOFAS. In the EPF group at follow-up the median function score (AOFAS) was 94 (range 78-97) compared to 92 (range 78-95) in the PRP group. However, due to the amalgamated result of assessment timepoints we were not able to extract an effect estimate for any time points of interest.

*Pain*

It was unclear which pain descriptor was assessed in this study, hence, we judged the outcome to be a secondary outcome. Both groups demonstrated improvements in pain over time; there were no between-group differences ‘over-time’ for VAS for pain. In the EPF group at follow-up the median pain score (VAS) was 2.35 (range 1-4) compared to 2.9 (range 1-4) in the PRP group. However, due to the amalgamated result of assessment timepoints we were not able to extract an effect estimate for any time points of interest.

Endoscopic plantar fasciotomy versus extracorporeal shockwave therapy.[26]

*Secondary Outcomes*

*Function*

In the EPF group at 3 months the median function score (AOFAS-AHS) was 77 (interquartile range 72-84) compared to 80.5 (interquartile range 73-85) in the ECSWT group. At 12 months the median function score (AOFAS-AHS) in the EPF group was 86 (interquartile range 76-89) compared to 87 (76.75-97) in the ECSWT group.

Lateral plantar nerve release with drilling versus lateral plantar nerve release without drilling.[27]

*Secondary Outcomes*

*Function*

The authors reported the group receiving calcaneal drilling had a better outcome at follow-up than those not receiving calcaneal drilling, however the exact post-surgical timepoint of the presented outcome is not clear, and no baseline measures were reported for the modified Mayo scoring system. We were not able to include results from this study in our analyses at predefined timepoints as their results reported an amalgamated result of assessment timepoints ranging from 25.6 (+/-14) to 27.6 (+/- 20.4) months for the two groups; hence we were not able to extract an effect estimate for any time point of interest.

Open plantar fasciotomy versus PMGR.[28,29]

*Secondary Outcomes*

*Function*

No clear between group differences were noted in the short (effect size [95% CI]: 3.70 [-3.42, 10.82]), mid- (effect size [95% CI]: 7.60, [-0.67, 15.87]) and long-term (effect size [95% CI]: 2.30 [-4.90, 9.50]) follow-up AOFAS-AHS scores.

*Pain*

It was unclear which pain descriptor was assessed in this study, hence the outcome was judged to be a secondary outcome. No clear between group differences were noted in the short (effect size [95% CI]: 17.57 [-0.92, 36.06]), mid- (effect size [95% CI]: -7.90 [-22.70, 6.90]) and long-term (effect size [95% CI]: -10.40 [-23.75, 2.95]) follow-up VAS scores.

*Health Related Quality of Life*

At twelve month follow-up, no clear between group differences were noted across all domains of SF36 (effect size [95% CI]: Physical functioning -2.60 [-10.44, 5.24], Physical role functioning 2.60 [-3.77, 8.97], Bodily pain 2.80 [-10.77, 5.17], General Health Perceptions 2.00 [-3.85, 7.85], Vitality 0.20 [-8.03, 8.43], Social role functioning -3.40 [-10.53, 3.73], Emotional role functioning -0.90 [-8.64, 6.84], Mental Health 0.90 [-7.16, 8.96]).

*Medication use*

Although no data were presented, the authors report that, in the first 15 days post-surgery, there was no between group difference in opioid (tramadol) consumption.

EPF with heel spur removal versus controlled non-operative treatment (corticosteroid injections).[30]

*Secondary Outcomes*

*Function*

No data reporting for outcome measures beyond the mean difference on FFI at 6 months was reported; instead, results were predominately presented graphically. Numerical data for 3, 6 and 24-month follow-up was made available on request following contacting the corresponding author.

No between group differences were noted in the short- (effect size [95% CI]: 19.14 [-9.02, 47.30]), mid-(effect size [95% CI]: -7.14 [-22.42, 8.24]) or long-term follow-up FFI scores (effect size [95% CI]: -5.06 [-10.91, 0.79].

*Pain – during activity*

No between group differences were noted in the short-term (effect size [95% CI]: 3.14 [-15.38, 21.66]). A lower (improved) VAS score was noted in the fasciotomy group at 6 months (effect size [95% CI]: -12.64 [-24.13, -1.15.]) and at 24 months (effect size [95% CI]: -11.73 [-19.17, -4.29]).

Endoscopic plantar fascia release versus cryosurgery.[31]

*Secondary Outcomes*

*Function*

Both groups AOFAS-AHS scores improved significantly at 12 months compared to baseline, with a greater improvement in scores at both 6 (effect size [95% CI]: 10.70 [3.63, 17.77]) and 12 months (effect size [95% CI]: 11.40 [3.86, 18.94]) for the endoscopic release group. No between group differences were noted in short-term follow-up AOFAS scores (effect size [95% CI]: 3.10 [-3.53, 9.73]).
