# Supplementary material for: What is the evidence for efficacy, effectiveness and safety of surgical interventions for plantar fasciopathy? A systematic review
Source: PLoS One. 2022 May 18;17(5):e0268512. doi: 10.1371/journal.pone.0268512 (PMC9116678; doi:10.1371/journal.pone.0268512)
Supplement: Attachment — Submitted filename: Response to Reviewers.docx [file pone.0268512.s009.docx]

Dear Academic Editor and Reviewers,

Thank you for your time in reviewing our systematic review entitled “What is the evidence for efficacy, effectiveness and safety of surgical interventions for plantar fasciopathy? A systematic review” and for the helpful feedback provided.

We have addressed each point raised and a summary of our responses can be found below:

Journal requirements:

Point 1: we have ensured that the manuscript meets the PLOS ONE style requirements. Any changes made can be seen within the uploaded file labelled 'Revised Manuscript with Track Changes'.

Point 2: we have updated our search up to February 25^th^ 2022 and made relevant changes within the manuscript and Figure 1 accordingly. Any changes made can be seen within the uploaded file labelled 'Revised Manuscript with Track Changes'. In summary we found no additional published trials, but the search did reveal a recently registered trial protocol.

Point 3: We have added further detail to the ‘Data Availability Statement’ further clarifying the exact location of the minimal data set underlying the results described, specifically that the minimal data set can be found within the document in Tables 1 and 2 in addition to that in the Supporting Information Files. If there is any further data that you would wish us to include please let us know.

“Data Availability Statement: The minimal data set underlying the results described can be found within the manuscript in Table 1 and 2, and within the Supporting Information Files.”

Point 4: The corresponding author has been altered to Neil O’Connell.

Point 5: New copies of Figures 2 and 3 have been uploaded.

Point 6: Captions for our Supporting Information Files have been added at the end of the manuscript, and in-text citations have been updated to match accordingly.

Point 7: The references list has been reviewed and in some cases references have been adapted to ensure they meet the PLOS ONE style requirements eg additional author names and DOI’s added. Any modifications made can be seen within the uploaded file labelled 'Revised Manuscript with Track Changes'. We did not find any references that had been retracted within the reference list other than an update to the NICE Plantar Fasciitis Clinical Knowledge Summary which we updated from 2015 to 2020.

Reviewers Comments:

Point 5:

In response to Reviewer #2, thank you for your comment regarding our reasons for including the Sadak study within our systematic review. The Sadak study was included in our review as their inclusion criteria (people with “resistant plantar fasciitis after ≥6 months of conservative management. The participants in their study had their diagnosis confirmed by the presence of a calcaneal spur and perifascial oedema, and increased plantar fascial thickness of >4 mm”) met our inclusion criteria (“studies involving adults, aged 18 years or older, diagnosed with plantar fascioapthy, or with an alternative diagnostic label for this condition e.g. plantar fasciitis, plantar heel pain. We included studies regardless of symptom duration or whether radiological diagnostic imaging had been employed”). Of note and of interest, even though they appear to have chosen a technique that may seem more indicated for people presenting with neural symptoms, such as Baxters neuropathy, the authors do not describe neural symptoms as an additional inclusion criteria for their study.

We thank you for your time in considering our revisions and comments and look forward to hearing from you.

Kind regards,

Sian

Sian MacRae PhD

Advanced Physiotherapy Practitioner
